# Practical Treatment of Mouth Pyorrhea

**Published:** 1910-12-15

**Authors:** D. D. Smith

**Affiliations:** Philadelphia


### PRACTICAL TREATMENT OF MOUTH PYORRHEA.

BY DR. D. D. SMITH OF PHILADELPHIA.

NOTE,—This paper embodies the discussion by Dr. Smith of a formal paper read by Dr. J. P. Buckley, of Chicago, before the First District Dental Society of New York, January 1910, entitled the Practical Treatment of True Pyorrhea. The paper was printed in the August, 1910 issue of the Dental Cosmos, but for some unexplained reason Dr. Smith's discussion has not been published. Because of the valuable data which it contains on the general subject we are pleased to present it to our readers as revised by the author.

Mr. President and Gentlemen of the First District Dental Society:

The time has been when Philadelphia was conceded to be the center of dental literature, dental education, dental appliances and dental culture. Latterly the glory seems to be departing from us and is being carried westward. Perhaps the most talked of, if not the most scientific, men in dentistry to-day are from what we call the West—the great city of big feet; we of the East will soon have to acknowledge that it is also the city of big brains for dentistry. (Applause.)

Witness the preparations being made to honor Dr. Black in the great convention and banquet in Chicago; no other dentist in this country has ever been so honored and perhaps none will be for years to come. Although you New Yorkers are inviting some of our older and better Philadelphians over here, ostensibly to *honor* them and incidentally to feast yourselves, I believe these occasions will all be greatly eclipsed when Dr. Black is given the contemplated banquet in Chicago.

Further exemplification of the tendency to recognize the West in dental matters is in evidence here this evening. Dr. Buckley has been called all the way from Chicago to read the paper to which we have just listened on the mystical subject of Alveolar Pyorrhea. Where could this society have gone except to Chicago for such a dissertation?

On some accounts I am glad I accepted your invitation to discuss Dr. Buckley's paper to-night and that I am here for that purpose; but if you will permit me, Mr. President, before taking up the discussion I would like to tell you why I am especially glad to be here. It is not because I wish to antagonize Dr. Buckley but for the reason that we heard those bewitchingly interesting "Incidents of Office Practice," at the outstart, especially that part relating to porcelain root ingrafting. I have often wondered how it could be that a porcelain root ingrafted into the crown of a molar, as we saw it so beautifully *pictured* in the *Dental Cosmos* some time ago, could be a help to any tooth either by increasing its attachment to the alveolus or by prolonging its usefulness. So I am glad to be with you,—as I have never seen one of these implanted roots, and I wanted to hear an explanation of the way in which it is all done. (I noticed one thing carefully, there was no attempt made to defend the process from the standpoint of utility.) We are told by the author of this complicated piece of dental mechanism, that the porcelain root is not designed to extend *into* the alveolar process, but that it hangs *exposed outside* the process. Now don't you think, gentlemen, we can all understand how very useful as well as ornamental such an appendage must be? (Laughter.) I had supposed that these porcelain roots were made to extend into the cavity left from the excision and extraction of a natural root, and that in some way kind nature, or a kinder Providence, enveloped them with a new pericementum, very likely providing a new lining membrane for the socket also.

I was mistaken in this, it seems, for these roots, however beautiful they may be on paper are never supposed to extend into the alveolar process. We are told they are not even indicated except in cases where the process in all absorbed. Having heard this I can more readily understand how they not only do no good but being admirable infection retainers, they much more quickly put the buccal roots of molars to which they may be attached out of service. I can also understand that they are of no possible use to the patient but that they do serve to enhance the general infection of the mouth and destroy the individual tooth more quickly and completely than would otherwise be the case.

I would suggest that the article on excision of natural roots and the substitution of porcelain, so ably sketched in two dental journals, be studied by the profession with a view to seeing what can be done by a dental artist, and then the scheme itself be relegated to the mental scrap heap as being a procedure, perhaps the most impractical and senseless ever devised in dentistry.

Turning now to Dr. Buckley's paper: We were told in the beginning that he did not propose to discuss the *pathology* of the condition which he calls Alveolar Pyorrhea and Pyorrhea Alveolaris, indiscriminately. He says it is his opinion that a mistake has been made by the profession generally in the past, in grouping too many of the diseases of the gum, pericemental and alveolar tissues, under one heading and calling them all pyorrhea. Would it not have been greatly to our advantage if he had told us what "pyorrhea" really is, so that we might not be so liable to this mistake in the future? I do not remember that he even stated definitely what he *believed* it to be. How are we to know what the "diseases of the gum, pericemental and alveolar tissues," are or what alveolar pyorrhea is unless we are authoritatively told. To make his story complete it seems to me the essayist should have told us this. It was Dr. Talbot who gave us "gingivitis" for pyorrhea, but I could never see that changing the name enlightens us in the least respecting the trouble. Dr. Talbot tells us he has written over ninety articles. I can believe him, and I hope he may live to write ninety more, all as good as his article on "Sense and Nonsense as Taught in American Dental Schools," published in *The Dental Brief* and in *The Dental Digest.* I want to say, aside as it were, that one of the most truthful things Dr. Talbot ever said is that

"dentists are not readers." Some of you may not like such plain talk, nevertheless I believe his statement is true. Dentists are not readers and I sometimes think it is just as well they are not readers whilst the chief purveyors of dental literature are the Commercial Journals, published mainly for advertising purposes. There are good reasons why dentists are not readers, but it is not my province to go into a discussion of that matter at this time. I have read *some* of Dr. Talbot's article with interest, but I have never yet been able to discover what this great "bug-a-boo," "gingivitis" is. I doubt very much whether there is a gentleman in the room who can tell with any certainty what Dr. Talbot means when he is talking about "gingivitis." How many can, with discrimination, diagnose a case of pyorrhea or define what mouth pyorrhea really is? Is it not then important, especially in the present undefined state of this trouble, that teachers should discuss the etiology of this condition?

It has long been a principle in surgery, as it should be an end sought in medicine, to first discover the cause of a trouble or disease and then eliminate it if possible. One great hindrance to the advancement of dentistry is that it is too superficial; it does not delve deep enough into causes. It seems all too readily satisfied with finding holes in the crowns of teeth and filling them. If we do not and cannot understand what mouth pyorrhea is, how are we ever to get at intelligent treatment for it? That there may be, as the essayist has said, mouth and gum troubles called pyorrhea, which are not pyorrhea at all, is quite true. That there are many cases of perfectly healthy mouths that have been called pyorrheatic by inexperienced practitioners in both medicine and dentistry, simply because of a little *normal recession* of the alveolar process and gum structure from the labial portion of some tooth-root, is also true; but that men *who ought to know* should write and talk upon the subject without even recognizing that alveolar pyorrhea is of necessity associated with a pus formation about the necks and roots of teeth, is incomprehensible.

Let us for a few moments take up that most important part which was left out of this paper and say something respecting the etiology or the real cause of mouth pyorrhea; for how can we with intelligence set forth directions for the treatment of it, more than for the treatment of any disease, unless we know the cause? First, I would like to ask whether mouth pyorrhea ever occurs in an edentulous jaw. Who ever saw a pyorrheatic condition in an edentulous mouth or in any part of a mouth where there are no teeth? Who dares to say he ever saw such a condition? I don't see any hands up. Is alveolar pyorrhea then a disease of the alveolar process? No, it is not. If it is not a disease of the alveolar process, is it a disease of the gums or other tissues of the mouth? Emphatically, No! If not found in the alveolar process when the process is devoid of teeth, it cannot be systemic. I have said in another place that we never have, *we cannot have*, alveolar pyorrhea disassociated from teeth in the alveolar process. The cause of this trouble must then of necessity be a tooth-root itself or some irritant associated with the root, giving rise to the pyorrheal inflammation. Alveolar pyorrhea, is therfore a pus discharge from about a tooth-root *in the alveolus*; it is the result of an inflammatory process induced by some irritant or abnormality associated *with a tooth* or its root; and it is certain that it never occurs except in such connection. Is not this true? The essayist says, "When I speak of pyorrhea alveolaris in this paper I mean true pyorrhea," and yet he leaves us wholly in the dark as to what "true pyorrhea" is. He tells us that "the true cause for this kind of pyorrhea, if there be more than one kind, has never been satisfactorily determined," and at the same time says, "prevention and failure to cure this condition is due to the "family dentist." That surely is not enlightening. We are all "family dentists" and we want to be instructed respecting the cause, the prevention and the cure of this trouble.

If alveolar pyorrhea is a constitutional disease, we want surely to know it, for then we must direct our efforts to alleviate or cure it through internal or constitutional treatment; if it is a local trouble our treatment for alleviation or cure must of necessity be local treatment.

A great many writers and speakers, some even here in New York, have endeavored to shake these unshakable enunciations. They have told us and tried to make us believe, that it proves nothing when we take a pyorrheatic tooth out of the mouth and cure the pyorrhea without further treatment. Now we know that it does prove something. "If we have gout in the toe," they say, "and we cut the joint out we cure the gout." But that is not true. All such talk is purely controversial and so lacking in physiological reasoning that I feel inclined to say, "Ephriam is joined to his idols; let him alone." There is no similarity whatever between the joints of the body in which gout occurs and tooth articulation. Rheumatic and gouty conditions arise in the *closed joints* always. They are the joints endowed with cartilage and ligaments and I suspect that it is in these tissues, especially the cartilage, that gout centers. The articulation of a tooth is wholly unlike the articulation of these joints; *it* is gumphotic (as a nail driven into a board); it is an open and exposed articulation, not a joint; it has no cartilage, no ligaments. The American Text-book of Operative Dentistry, one of the most unsatisfactory compilations in modern dental literature, considering the time of its advent speaks of "the *ligament*" of a tooth. Who ever saw "the ligament" of a tooth? There is no such thing. The nearest approach to a ligament connected with a tooth is in those cases referred to by the essayist where a loosened tooth, generally a molar, will sometimes be found with the bony attachment wholly necrosed. Occasionally such a tooth will be held in its socket by what appears to be a kind of ligament, really hypertrophied gum structure, attached to a limited area of the cervix; the looser the tooth becomes, the more nature seems to toughen and render ligamentous this section of the gum, the only structure which is holding the tooth in place.

This apparent *ligament* is wholly abnormal. I have in no case seen these loose molars held by this ligamentous gum attachment where the pulp was not alive.

The essayist speaks of the benefit of devitalizing pulps for pyorrhea. What real benefit can come from destroying a pulp for an inflammatory attack upon the pericementum? Certainly there could be no possible justification for destroying a pulp, or making any other attempt to save a tooth in the condition described.

If I have not made clear the fact that pyorrhea is *not* a constitutional trouble, let me ask you to extract your pyorrheatic teeth and see how quickly the pyorrhea disappears, the cause of it being removed. Extraction always cures the pyorrhea and it were infinitely better to lose the tooth than that the pus discharge in the mouth should be continuous.

Point, if you can, to a single instance, in which the "constitutional" man ever suggested a remedy with any definiteness. (Pardon me, I did once hear one of your New York men suggest a sea voyage for it.) I think it is not unlikely that the supremacy of Philadelphia as a center of dental education began its decline when one of her prominent men declared that this trouble was caused by "uric acid" and "gout." It was certainly about that time. It seems that the profession in Philadelphia struck twelve with that declaration and what followed it and it has been on the toboggan ever since.

The profession, at the time, without chart or compass respecting "Riggs' disease" were keyed to follow any scout or adopt any theory that might present and so the leaders, themselves wholly ignorant of the true nature of the trouble, proclaimed it "a constitutional disease due to gout." This proved quite popular amongst the laity for a time, as "gout in the teeth" was thought to have quite an aristocratic sound. The author of the "American Text-book," lent his influence to the constitutional side and cited Munkowsky as "throwing light upon it," (mouth pyorrhea) when he extirpated the liver of an old goose and found that "thereafter he excreted, not uric acid, as in the case of a normal goose, but that he excreted large quantities of lactic acid and ammonium in *quantative relations of ammonium lactate.*" That has something of a scientific sound and I trust, gentlemen, you see "the light thrown by it upon mouth pyorrhea." (Laughter.)

The discussion of pyorrhea as a constitutional disease by medicine and dentistry literally opened the flood gates of utterly worthless commercial preparations and set the profession wondering. Write, gentlemen, "better not" on the label of every offering as an internal remedy for mouth pyorrhea. Uric acid and gout have just about as much to do with the development of pyorrhea as pericardemia has to do with a sebacious tumor of the scalp, not more.

To show the absurdity of these constitutional theorists,—about six years ago there came to me a very bad case of pyorrhea from one of the largest and most fashionable practices in Philadelphia. The gentleman was suffering almost to the limit of human endurance. He had been for four years under treatment vibrating, as he affirmed, between the dentist's office and his physician's office, but without permanent relief. His dentist told him that he had "gout in his teeth and he must be treated medically," etc. He was accordingly advised to go to his family physician to be treated for gout and rheumatism, troubles that he knew nothing of as he had never had a gouty or rheumatic pain in his life. The physician cut out roast beef from his diet and told him he would be better "when the strawberry season was over." Here was a turning of the case over to the "family physician" as the essayist has advised doing this evening. How very convenient such a procedure! How happily it covers ignorance and incapacity! What does the family physician know about pyorrhea or other mouth conditions? Did he ever look into the mouth except to examine the dorsum of the tongue? Did you ever hear a medical man suggest anything regarding mouth conditions, or of there being any causes of disease in the mouth? Not often, I think.

Time will not permit my following out in detail treatment of the case cited. Examination disclosed a chronic case of alveolar pyorrhea, complicated with a long standing pericemental abscess on an upper molar; an empyemic antrum attended with nasal pus discharge; several loose teeth including two bicuspids and an upper central, together with general mouth infection. Think of this man, an army officer, suffering intensely as he had done at times for four years, being treated for gout and rheumatism with colchisum and a dietary which excluded roast beef and strawberries! Extraction of the molar with the pericemental abscess (the only treatment for a tooth with this form of abscess) together with *local* remedies, alone brought entire relief in three days with marked diminishing of nasal discharge on the sixth day. In three months from beginning treatment the pyorrhea was cured, the pyorrheatic teeth were firm in their sockets and four small pieces of bridge work restored the arches to perfect condition. An opening was made through the alveolus bucally, into the antrum and kept open with tent for over a year, when it was allowed gradually to close. This operation is now over six years old, the prophylaxis treatment has been maintained with regularity from the beginning. The health and appearance of the mouth is perfect; there has been no distress, no discomfort.

If there seems to be a constitutional taint in some cases of pyorrhea behind the local causes, it is because we have not yet comprehended the local conditions.

The paper apparently reluctant to deal with the etiology of pyorrhea, is based on the assumption that there is always something at the roots of teeth causing it. Look at this array of instruments exhibited here by the essayist. What are they for? Are they not for removing something from the teeth and roots? Throughout the whole paper, if I understand it, the writer has in mind calcareous accumulations *to be removed.* Something to be removed most undoubtedly.

There is a great deal of calcareous matter on teeth which is not productive of pyorrhea and which does no special injury to the teeth themselves. There are cases where masses of tartar adherent to the teeth produce most disgusting conditions of the mouth; this of course should be removed and the teeth subjected to the prophylaxis treatment; but these conditions are not necessarily productive of pyorrhea. They may indeed exist without great injury.

What then are some of the more prolific causes of mouth pyorrhea? Elsewhere I have spoken of it a "disease of filth" and there is little occasion to modify this assertion for if the mouth and necks of the teeth be kept positively clean by frequent and thorough change of environment and the teeth given the prophylaxis treatment, there will be no pyorrhea. The most important point in the paper is where it emphasized local treatment to prevent development. That is what dentistry has not yet done; it has yet to learn to so treat the mouth from childhood and so to keep it in condition, as to prevent the development of pyorrhea in adult life, for pyorrhea develops almost exclusively in adult life. (Applause.)

The paper said some things very well in regard to the treatment of pyorrhea, but it omitted also many things of importance.

What are some of the conditions that cause pyorrhea? Mouth pyorrhea, I repeat, is a disease, if it can be styled a "disease," of uncleanliness. I do not like the term "disease" applied to it, for mouth pyorrhea is really a local inflammation resulting in pus discharge. It comes largely from neglect *on the part of dentistry.* Dentistry has never given the matter any attention whatever until very recently. It has held cavities of decay so close to the eye, that it has not been able to see with certainty anything else in the mouth.

Among the causes of pyorrhea there should be mentioned the *size, shape and arrangement* of the teeth in the mouth; but more than all, lack of care on the part of the patient and dentist. Temperament also has something to do with it. Pyorrhea never develops in a purely sanguine temperament. It occurs in the bilious, the lymphatic and the nervous temperaments and many compounds of these, but never in the sanguine temperament.

We place emphasis on these conditions of the mouth and teeth as causes of pyorrhea, because they interpose great obstacles to self care on the part of the patient and interfere greatly with the cleanliness of the mouth. Conditions of the teeth themselves, their shape, often large bell-shaped crowns with constrictions at the necks, accompanied with irregular arrangement in the arches, contribute markedly to the induction of pyorrhea and to pyorrheatic conditions. The short regular crowns of the sanguine temperament in which there is no constriction at the cervix do not develop pyorrhea.

Cemental conditions also have much to do with the induction of pyorrhea. There is a cementum on some teeth that becomes denser with age, and changes practically into dentin as the tooth advances to and beyond maturity. You will see these teeth in the front of the mouth, especially the cuspids; and speaking of medical men understanding or not understanding this matter of pyorrhea and mouth conditions, I have in a number of cases found medical men diagnosing slight recession on a cuspid or a bicuspid on the labial and buccal faces of teeth as pyorrhea in mouths that are in perfect and normal condition. What then is the cause of recession in such cases? Simply that the cementum has been solidified and hardened into a structure which is almost indentical with the dentin with the result that there is not sufficient vitality in the thin layer of cementum at this point to maintain the pericemental life and it withdraws to the thicker and more vital territory. The teeth do not loosen in such cases; the gum structure and alveolus remain in perfect condition of health. There is no pyorrhea neither anything to excite apprehension. It cannot be expected of medicine to know matters like this but *dentistry ought to know*. There are other conditions mostly in the lower jaw, where pockets come quite rapidly, due to toxic matter remaining undisturbed at some inaccessible point on the neck of the tooth until inflammation results. This is followed by necrosis and pus discharge and final loosening of the teeth. This is true pyorrhea. It is to be combatted with persistent and intelligent local treatment and by this I do not mean a great array of dental instruments.

I have heard that there is a man, he must be a Chicagoan, I am sure who has one hundred and fifty four instruments in a set. Such a display of instruments, for it is naught but a display, will do far more to retard the treatment of pyorrhea than Dr. Buckley's paper can do to advance it. We want instruments to treat pyorrhea, but we must have common sense behind them. Possibly some day we may find a tartar solvent that will be of value, but for the present the best tartar solvent is the instrument that will remove whatever foreign matter may be found on the teeth and roots or in conjunction with them. We need to know that infection causing pyorrhea is not all calcareous matter.

The most deleterious pus inducing infection in connection with the teeth and roots is the gelatinous semi-solids, generally susceptible of removal with the porte polishers charged with the orange wood points and pumice. Some man in Boston, at a meeting of the Massachusetts State Society two or three years ago, affirmed that Dr. Smith cured, or professed to cure pyorrhea by "polishing enamel." Think of it ye men of intelligence! Could any man but a wilful misrepresenter make a statement like that after reading the articles I have written? If I should happen to be so shockingly misrepresented in what I say here to-night, I am sure some one in this audience would stand up and say "that is not true."

Dr. Buckley's paper inferentially rather than positively gave assent to the proposition that the cause of pyorrhea was local and that local treatment is required to cure it. Pyorrhea can be cured; but it must not be ssumed that it can be cured and at the same time retain *all* teeth regardless of conditions. It can be certainly and quickly cured by taking out any and all teeth infected, and that without further treatment; but if it is desired to retain the teeth, attention must be directed to such as have some mechanical support in the alveolus.

When a tooth has lost its bony support through necrosis and absorption of the alveolar structure, there is no such thing as restoring that tooth to usefulness. Though a tooth may be very loose, the cure of pyorrhea may be affected in the sense of stopping the pus discharge but not always in the sense of restoring the necrosed alveolus. To treat pyorrhea with a view of benefitting the patient, all teeth with pericemental abscess, and all that can be rotated in their sockets, even to a slight degree, as well as those that have lost alveolar support, should be extracted in the beginning.

It was suggested by the paper that in some cases there is a constitutional treatment which might be resorted to with benefit. I take issue squarely with that proposition. It can not be done. Whoever heard of one of our constitutional theorists really recommending any constitutional remedy for pyorrhea. They talk and write of "appropriate constitutional remedies for pyorrhea," but who dares to designate these remedies? Did Dr. Buckley? There is nothing of a constitutional nature that can in any way benefit mouth pyorrhea except so far as constitutional medication may be directed to the improvement of impaired general health.

I have yet to know of a single dental writer that ever ventured to commit himself to a line of constitutional treatment for pyorrhea. The so-called remedies put out by the commercial houses, some of them very cleverly and insiduously advertised, are each and all utterly valueless in the treatment of this disorder. All talk of instituting constitutional treatment for mouth pyorrhea by dentistry is the merest "buncombe."

Now one word in regard to the terms prophylaxis and prophylactic, they ought not to be used interchangeably as in meaning they are essentially different. Prophylactic is a remedy; prophylaxis is an operation. Let us make the distinction and we will always understand the meaning.

The paper suggested the correction of the occlusion of teeth as one of the remedies for pyorrhea. That was advocated by Dr. Bonwill years ago. If the essayist were not present to hear me I should say decidedly this is a mistake The correction of the occlusion of teeth has nothing to do with the cure of pyorrhea. You can never arrest the movement of a displaced, twisting, pyorrheatic tooth until you arrest and cure the inflammatory process which is forcing it out of position. Be not deceived; truth is not mocked.

I want to say a word respecting the mechanical tightening of teeth loosened from pyorrhea. Tightening teeth by tying them to others by means of grass strings or other means is but a temporary expedient; all devices of this nature obstruct cleaning, favor the retention of infection and hasten the loosening of the good teeth used as supports. It is an operation not to be encouraged. Loose teeth can sometimes be tightened permanently and to advantage; they can be held in place by a fixture that is efficient, permanent and perfectly cleanly; but this cannot be said of any string fastenings. In February, 1902, I read a paper before the New York Odontological Society, entitled, "An Original System of Tightening Loose Lower Incisors and Cuspids," Embracing a Method of Securing Substitutes Without Clasps or Plates When These Teeth are Missing." The paper is illustrated and gives full details of a most satisfactory appliance for securing loosened teeth in all parts of the mouth, but I cannot rehearse the matter this evening.

I want here to say a word about a new method of treating certain cases that I have been using more recently. I consider it a very valuable adjunct in the treatment of certain pyorrheatic conditions. First, in regard to the restoration of pockety teeth;—if there is a deep bad pocket between two molars, as between the mesial root of a second molar and the distal root of a first molar, with the process and gums *between the roots* of both molars in good condition the prognosis is favorable even if one or both teeth be loose; but if we have a similar condition of pocket *between the roots of one or both teeth*, you have a far more problematic condition to treat and a prognosis less favorable. Keep your eyes open. In the one case where the pocket is between the teeth you may make headway in treatment, while possibly in the other, where the necrosis is between the roots of the same tooth the case will go on to complete destruction.

Let me illustrate this on the blackboard; I am very poor at drawing, but I can make something to assist in presenting more clearly what I want to say. Now, I want you to note carefully that what I am describing is all confirmatory of the local origin and perpetuation of pyorrhea. It is a method of treatment for certain cases that I have adopted in the last two years. It grew out of finding cases of bridge-work where the gold crowns on molars especially, would cause necrosis of the process at the bifurcation of the roots whether on the upper or lower jaw. The opening between the roots sometimes becomes exposed below or above the crown, causing rapid infection and creating a pocket. I have seen many bridges ruined by gold crowns in connection with molars, acting in this way. Many *single* crowns have been lost from the same cause. Can this be prevented? Convinced that if this infection between the roots especially of molars could be barred out of the necrotic cavity the tooth could be saved, I have in many instances cut the gold cap down and made a positive retentive cavity *between the roots* for filling. Such cavity may be between the roots of a lower molar or between the buccal or the palentine and buccal roots of an upper molar. I have filled these cavities solid with the best of the hard gutta perchas, forcing it whilst soft, hard against the process and gum, leaving it flush with the, alveolus and gum. In every case, and I have performed this operation in a great many cases, I have found this gutta percha filling to arrest all recession, to be absolutely non-irritating and to harmonize perfectly with the living tissues. The result has been the keeping out of all infection, and the retightening of the tooth. I recommend it to you.

One thing further before I sit down. I take issue with the paper in regard to the use of chlorides. The essayist tells us that the chlorides are never to be used in the mouth. I do not know exactly what he means, but I tell you, you will never make great progress in treating mouth pyorrhea without the use of Zinc Chlorid. Some of you have been in Philadelphia and have seen some of the mouths we have presented. Let me here give you all the medicaments used in those cases. They were not treated with prophylactics or any *internal* medicaments whatever. But I do use Chlorid of Zinc as a local application and it is the grandest remedy ever found for pyorrhea. Whatever the pyorrhea, acute or chronic, Zinc Chlorid, not the crystals, but deliquesced Chlorid of Zinc, diluted just enough to give it fluidity should be applied in all pockets, carrying it to place on an orange-wood point or a wooden toothpick, never a metal point; dip the *orange-wood point* into the liquid and carry it directly into all pockets and see how quickly they will begin to change in appearance and the tooth begin to tighten. Thorough cleaning and polishing should precede all medication. I do not mean to say that pus pockets will close immediately or after one application, but new structure will begin to form in due time and little by little (for treatment must be continued) healthy granulations will be thrown out and the teeth tightened just so far as the wasted alveolar tissue will permit I do not look for miracles in the restoration of necrosed and wasted tissue, but I do look for eradication of pyorrhea; a reformation of the alveolar and gum structure; I look for all tissues about the teeth to take on a perfectly healthy condition. In connection with this I hardly dare tell you the local applications I use, for some of them being secret, you may not regard them as *strictly ethical.* Zinc Chlorid which I have so happily used for a number of years is pharmacopeal, therefore no doubt can arise respecting it, but when I tell you of the other remedies I use I may have to apologize. I heard a distinguished surgeon of Philadelphia once say of paregoric, behold "a God-given remedy." Permit me to say in this connection that "Phenol Sodique" and "Zhongiva" should be regarded as God-given remedies in dentistry. If they are not "ethical", they are certainly efficacious. *You* apply the Zinc Chlorid and *you* alone apply the phenol-sodique, but the patient should be required *to use Zhongiva.* Any dentist who attends strictly to the details of the prophylaxis treatment can produce with these applications as good results as my friend here, who is giving himself wholly to this work, or as Dr Buckley, who has given us this interesting and valuable paper to-night. Let it be remembered that Zinc Chlorid is to be applied on *wood points in all pyorrhea pockets*; the phenol-sodique *used only by the operator,* is to be applied freely to the gums immediately after operating, and Zhongiva, a most important remedy, should be used at least twice daily by the patient, immediately after cleaning the teeth.

